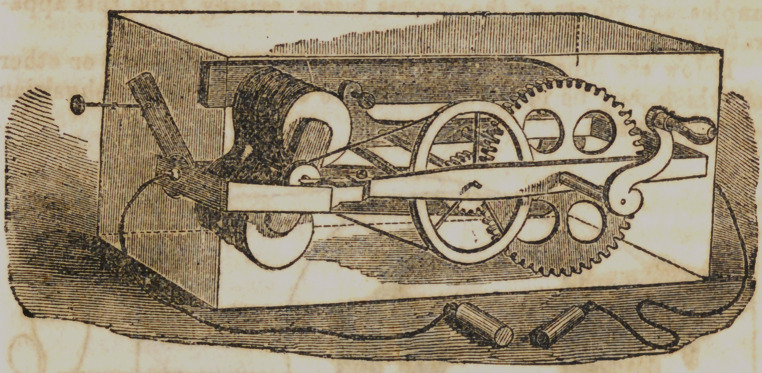# Extracting Teeth by Galvanism

**Published:** 1858-08

**Authors:** 


### EXTRACTING TEETH BY GALVANISM.

This subject is now attracting considerable attention, the dread of pain being instinctive and universal, every thing which holds out any plausible means of avoiding suffering, is embraced with avidity. Ether, chloroform, local anasthesia by freezing, each have had their day of popularity, and last of all comes galvanism as *the perfect thing*, an old principle applied to new uses, and "*patented*" of course!"

"The apparatus necessary is the ordinary magnetic or galvanic battery, the negative pole of which is attached to the extracting forceps by means of a hook at the end of the tissue cord or wire, which is made to pass through a hole drilled in one handle of the forceps, so that the electrical current shall pass from the tooth through the forceps and wire or cord to the instrument. The other pole of the battery is connected with the patient by means of another cord or wire and a metallic handle which completes the circuit as soon as the forceps come in contact with the tooth or gums.

"It is well to have the cord or wire from the machine to the hand *interrupted*, but so arranged by means of a foot-board, that the circuit can be completed by pressure on the foot-board, and that the current be *not* allowed to flow until the forceps grasps the tooth ready to be extracted. As it has been observed that the current does not paralyze the sense of pain, when it has been applied a little length of time. Only the shock of its application and the *immediate* loss of sensation seemed to answer the purpose completely.

"The hand of the operator which grasps the forceps, should be clothed with a silk or kid glove to prevent his receiving a part of the current, and the one which removes the cheek from the tooth, should also be protected by the folds of a napkin.

"One of the Committee appointed by the Franklin Institute, says: 'Most persons suppose there is a *shock* experienced on its application, or that it is painful to the tooth; these objections are not correct.

"'We applied it in many cases and the pain was considerably less than in the ordinary way, in all except one case.' * * 'Some patients ask, when they observe us leave the mouth, whether we have taken out the tooth or not, as they are not sensible when the tooth left the socket, although they know that we had put the forceps on the tooth, and that an effort at extracting had been made.'

"The Committee report at length, and from that report we make a brief extract.

'One hundred and sixty-four teeth were extracted in the presence of the Committee.

'The Committee is satisfied, from the observation and experience of its members, that in a large majority of cases of extraction with this apparatus, *no pain whatever* is felt by the patient. * *

'To test the question whether the effect might not be simply mental, the circuit was broken without the patient being aware of it, when the usual pain was experienced, although, in the same patient, and on the same occasion, teeth had been removed while the current was flowing, without causing pain * *

'The sensation produced by the passage of the current is not painful, it being so adjusted as to be *just perceptible* to the patient. The Committee believes its use to be entirely without danger, and not likely to be followed by any unpleasant after effects. * * * The operator requires no new instruments except the battery and coil. * * * *

'As to the theory of these very singular and unexpected results, the Committee does not express an opinion; of the facts it is fully satisfied.'"

In the Southern Medical and Surgical Journal for June, D. S. Chase, M. D., D. D. S., relates his experience to the following effect:

"The *First Case* in which I tried it, I removed seven teeth, all firmly set—five molars and two cuspidati or eye-teeth. In extracting the first tooth, too much electricity was applied, and the patient complained of pain from the shock, but not from the removal of the tooth. In the second tooth too little was applied, and the tooth itself gave pain. After this we were able to regulate the quantity, so that neither the electricity nor the extraction of the tooth gave much pain. Patient not at all nervous, and frequently expressed herself highly pleased with the operation. The feeling experienced during the extraction of the teeth, as she expressed it, was a benumbing sensation about the tooth, which appeared to be attached only to the gum.

"*Second Case.*—Extracted six teeth. Patient somewhat debilitated from previous suffering with her teeth, and quite nervous. Suffered considerable pain during the operation, but would not allow one to be extracted without electricity.

"*Third Case.*—Extracted four teeth. Patient suffered but little pain.

"*Fourth Case.*—Extracted a molar tooth, that had been previously broken, for a highly intelligent gentleman from a neighboring village. He was much pleased with the operation, and was very enthusiastic in his praises of electricity as applied to Dental Surgery.

"*Fifth and last Case*, that I will report at present.—Extracted *ten* teeth for an elderly lady. Expressed no fear or pain during the operation, and seemed to treat the affair as a mere trifle, which might be attended to any morning, without much inconvenience.

"The general expression by those who have tried it, seems to be decidly in favor of electricity in extracting teeth.

"In some of the cases mentioned above, the gums were lanced by the same process, by connecting the pole of the battery with the handle of the lancet, while the patient held the other—the hand of the operator being protected by a silk glove."

As before remarked, the ordinary magnetic or galvanic battery, or electrical machine, can be used—except Groves' Battery, which is inadmissible on account of the deleterious and unpleasant effects of the nitrous fumes arising from this apparatus when in use.

Below are illustrations of those in common use, one or other of which may be found in the office of nearly every physician in the country.

Fig. 1 represents "*Pike's Vibrating Magnetic Instrument for medical use.*" In this arrangement the armature vibrates between the platina points and one of the poles of magnet, the armature being in the form of a small hammer attached to a silver spring, having a platina center on that part in contact with the point, and the end supported from one of the poles of the electro-magnet, the hammer vibrating between the other poles and the platina point.

Fig. 2 is *Pike's Portable Magneto-Electrical Machine for medical purposes.* The machine consists of a double coil or helix, of coarse copper wire insulated over which is wound about two feet of fine insulated copper wire, in the interior of which is placed a bundle of soft iron wires, and which, when the machine is in operation, become powerful magnets, and regulate the strength of the power according to the extent they are placed in.

Fig. 3 represents *Davis' & Kidder Patent Magneto-Electro Machine.* At each end of this machine are the conductors, at the farther corner of the box on the left, is represented a sliding rod, which controls the position of the straight armature, in relation to the poles of the magnet, by removing this on or off the poles the strength of the shock may be regulated.

There is, however, a very neat and convenient battery recently got up, differing in some respects from all of the above.

Those, therefore, who have no apparatus on hand that will do, should of course, when obliged to purchase, take care to get the most approved kind, embracing the latest improvements, &c. Such an apparatus can be had for from $12 to $15. The ordinary electro-magnetic machines are sold at $10.

One of the most disagreeable difficulties to be overcome by the practitioner in tooth-extracting, is the *fear*, *dread*, or *terror* of the patient, and the use of the electrical current, in most cases does away with this completely, and patients quietly submit to have tooth after tooth, or frang after frang removed, with a degree of placidity and unconcern that is greatly to the relief of the operator.

The *exhaustion* or nervous depression which often follows tooth-extraction without the use of an anæsthetic, does not obtain when the galvanic apparatus is used; and there is no fear on the part of the patient or operator, which has so often prevented the use of chloroform and other anæsthetics.

We are informed that agents are already canvassing the country for the sale of rights. We regret exceedingly that a discovery from which the happiest results are anticipated should be restricted in any way. We learn that many are experimenting regardless of the patent, and we think the owner of the patent will find that they are engaged in rather unprofitable business. They may annoy some, but nine-tenths of the profession will use it without a right—except that right which Almighty God and the spirit of our Republican institutions grants to every man—the pursuit of happiness, the acquisition of knowledge, and the *use* of such knowledge. We understand that an application is now on file for the use of "Cold Water as a Beverage." This is equal to the tyrany of the people of Niagara, where they charge a *quarter* for the privilege to look at the sun! JEROME.

## Figures and Tables

**Figure f1:**
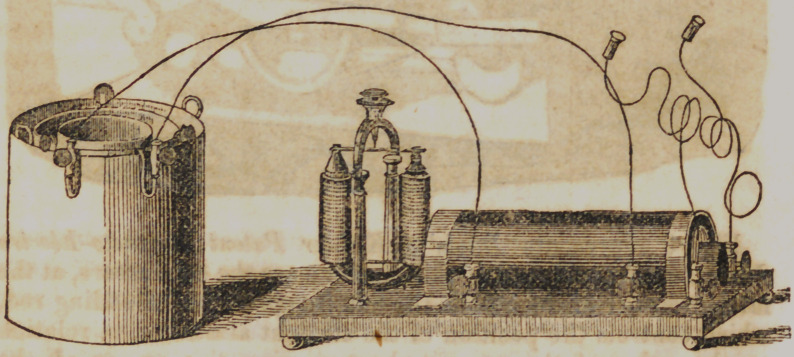


**Figure f2:**
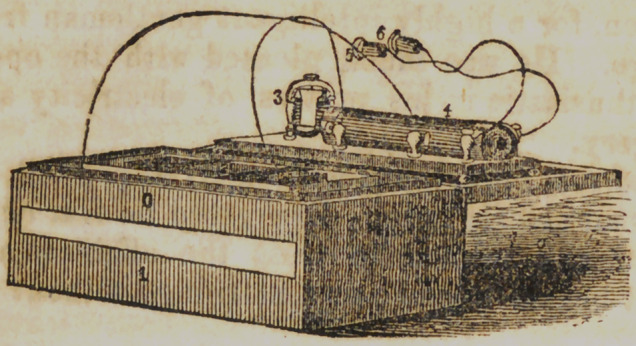


**Figure f3:**